# A phenolic ester from *Aglaia loheri* leaves reveals cytotoxicity towards sensitive and multidrug-resistant cancer cells

**DOI:** 10.1186/1472-6882-13-286

**Published:** 2013-10-27

**Authors:** Else Dapat, Sonia Jacinto, Thomas Efferth

**Affiliations:** 1 Department of Biology, University of the Philippines, Manila City, Philippines; 2 Institute of Biology, University of the Philippines, Diliman, Quezon City, Philippines; 3 Department of Pharmaceutical Biology, Institute of Pharmacy and Biochemistry, Johannes Guttenberg University, Mainz, Germany; 4 German Cancer Research Center, Heidelberg, Germany

**Keywords:** *Aglaia loheri*, Cytotoxicity, Multi-drug resistance, Apoptosis, JC-1 mitochondrial membrane potential, Annexin V-FITC

## Abstract

**Background:**

Bioactivity-guided fractionation of extracts of *Aglaia loheri* Blanco (Meliaceae) yielded a cytotoxic isolate, termed Maldi 531.2[M + H]^+^. This phenolic ester was further investigated for its *in vitro* cytotoxicity toward human CCRF-CEM leukemia cells and their multi-drug resistant (MDR) subline, CEM/ADR5000. The intrinsic mitochondrial membrane potential (ΔΨm) and induction of apoptosis by this isolate were evaluated.

**Methods:**

Chromatography techniques, mass spectrometry and proton NMR were employed to isolate Maldi 531.2[M + H]^+^. XTT cell proliferation and viability assay was used for cytotoxic test, and JC-1[5’,5’,6,6’,-tetrachloro-1,1’,3,3’-tetraethylbenzimidazoyl carbocyanine iodide was used to assess ΔΨm and initiation of apoptosis; Annexin V/FITC-PI staining was employed to analyse apoptosis.

**Results:**

Maldi 531.2[M + H]^+^ was cytotoxic towards both CCRF-CEM and CEM/ADR5000 cells with IC_50_ values of 0.02 and 0.03 μM, respectively. The mitochondrial membrane potential (ΔΨm) of MDR cells was significantly reduced in a dose-dependent manner leading to apoptosis as detected by flow cytometric Annexin V-FITC/ PI staining.

**Conclusion:**

Maldi 531.2[M + H]^+^ may be a potential anti-cancer drug candidate whose mode of action include reduction of the mitochondrial membrane potential and induction of apoptosis.

## Background

Hematologic malignancies account for a considerable percentage of cancers worldwide [1]. A common type of this malignancy is acute lymphoblastic leukemia (ALL). ALL involves rapid transformation of a single normal progenitor cell [2] triggered by several factors associated with exposure to radiation and chemicals. These factors cause genetic damage, resulting in complete destruction of cellular regulatory controls on proliferation, differentiation and apoptosis [3-6]. Since leukemia is a systemic disease, its cure depends on chemotherapy [7] rather than surgery. A new approach to increasing effectiveness to chemotherapy includes the use of various molecularly targeted drugs derived from natural plant products [8].

The research on *Aglaia loheri* was part of a larger project to explore the potential medicinal value of plants endemic to the mountain area of Kanawan, Morong (Bataan, Philippines) which is part of the ancestral domain of indigenous people called Aetas. From personal communications with the indigenous people, it was learned that some of the plants were used by the Aetas for medicinal or nutritional purposes. *A. loheri* is actually part of their diet. However, due to accounts from published literature on other *Aglaia* species, we hypothesized that *A. loheri* may be cytotoxic towards cancer cells.

The genus *Aglaia* (family Meliaceae) is an important source of unique bioactive natural products, which contain a cyclopenta[b]tetrahydrobenzofuran skeleton and include more than 50 naturally occurring derivatives collectively called rocaglamides [9]. This group of compounds was found to be effective against thymidine kinase-deficient *Herpes simplex* virus type 1 (HSV-1) and phosphonoacetate-resistant HSV-1 strains [9], including human amoebiasis caused by *Entamoeba histolytica*[10]. These compounds were shown to inhibit necrosis factor (NF)-B induced gene activation in human T cells [11], HIV-1 infection [12], and initiation and promotion of chemically-induced cancer on mouse skin [13]. Amide esters are structurally related to bisamides and flavaglines from *Aglaia sp.*[14] and exhibited anti-fungal [15], insecticidal [16], anti-tuberculosis and anti-viral activities [17]. Remarkably, this class of compounds also induces apoptosis in resistant human cancer cells [18-20]. For example, silvestrol, a novel compound from *Aglaia* was active against cancer cells *in vitro* and *in vivo*[20,21]*.* It induced apoptosis in prostate carcinoma cells through the mitochondrial/apoptosome pathway without activation of caspase-3 or −7 [19] and in human B-leukemia cells, by reducing Mcl-1 expression due to inhibition of translation with subsequent mitochondrial damage [21].

In the present study, crude extracts from leaves of *A. loheri* Blanco were subjected to bioassay-guided isolation by means of various chromatography techniques. The resulting active principle was further analyzed and characterized by mass spectroscopy and nuclear magnetic resonance (NMR). The isolated active principle was investigated for its cytotoxicity towards cancer cells. The mitochondrial membrane potential (ΔΨ_m_) was analyzed as a key indicator of cell viability [22,23] and induction of apoptosis as an important parameter of cell integrity.

## Methods

### Kits and reagents

Analytical grade ethyl acetate and hexane were used for extraction. Analytical grade chloroform and methanol were used for gravity column chromatography. Silica gel 60 G 0.063-0.200 mm (Merck; Germany) was used for gravity column chromatography. Pre-coated gel 60 G F_254_ plates 0.25 mm thick (Merck, Darmstadt, Germany) were used for thin layer chromatography (TLC). Iodine crystals and UV were used to visualize separation monitored by analytical TLC. Doxorubicin was purchased from Sigma Chemical Company, USA. Dimethyl sulphoxide (DMSO) (Sigma, St. Louis, MO, USA) was used to dissolve the test samples. 3-(4,5-dimethylthiazol-2-yl)-2,5-diphenyltetrazolium bromide (MTT) were purchased from Promega, USA. XTT (2,3-bis- (2-methoxy-4-nitro-5-sulfophenyl)-2H-tetrazolium-5-carboxanilide), XTT labelling reagent (sodium 3’-[1-phenylaminocarbonyl)-3,4-tetrazolium]-bis (4-methoxy-6-nitro) benzene sulfonic acid hydrate) and electron-coupling reagent (N-methyl dibenzopyrazine methyl sulphate [0.383 mg/mL (1.25 mM)] in sterile phosphate buffered saline (PBS) were purchased from Roche (Mannheim, Germany). JC-1 and annexin V-FITC detection kit was obtained from eBioscience (Frankfurt, Germany); propidium iodide and carbonyl cyanide 3-chlorophenylhydrazone (CCCP) from Sigma-Aldrich (Taufkirchen, Germany).

### Cell culture and supplements

Human colon cancer cell line (HCT116) was obtained from American Type Culture Collection (ATCC, Manassas, Virginia, USA). The cells were grown in McCoy’s 5a modified medium (Invitrogen, Carlsbad, CA, USA) supplemented with 10% inactivated fetal bovine serum (Invitrogen, USA) and 1% penicillin-streptomycin (100 U/mL) (Invitrogen, USA). The cell line was maintained in a humidified incubator containing 5% CO_2_ at 37°C.

Human leukemic cells, CCRF-CEM and their multi-drug-resistant subline, CEM/ADR5000 [24], were obtained from Dr. Axel Sauerbrey (Department of Pediatrics, University of Jena, Germany). The cells were maintained in a humidified environment at 37°C and 5% CO_2_. The cells were grown in RPMI 1640 [−] L-glutamine containing 1% penicillin (10 U/mL), streptomycin 10 μg/mL, and 10% heat-inactivated fetal bovine serum (FBS) (all obtained from Gibco Invitrogen, Germany). Drug resistance of of CEM/ADR5000 cells was maintained by weekly treatment with 5000 ng/mL. P-glycoprotein expression without overexpression of other ATP-binding cassette transporters in CEM/ADR5000 cells has been reported [25,26].

Peripheral blood mononuclear cells (PBMC) were isolated from freshly collected whole blood sample using Ficoll-paque solution (Histopaque-1077) (Sigma) by centrifugation [27]. The cells were washed twice with RPMI-1640 and were re-suspended in same culture medium (RPMI-1640). Cells (0.5 mL of cell suspension) were seeded in a 24-well sterile plate at a density of 10^6^/mL prior to activation with 0.5 mL of 10 μg/mL of phytohemagglutinin (PHA). Addition of 0.5 mL of PHA into each well (0.5 mL RPMI-1640 to one control group) adjusted the number of cells to a final density of 5 × 10^5^/mL per well. The cells were incubated at 37°C and 5% CO_2_ for 3 days prior to XTT assay.

### Collection of plant material

Fresh and mature leaves of *A. loheri* Blanco were collected in the morning from its natural habitat in Morong (Bataan, Philippines). Voucher specimen of the plant under Accession No. 14612 was deposited and authenticated by the Jose Vera Santos Herbarium of the Institute of Biology, University of the Philippines (Diliman, Quezon City, Philippines). The leaf specimens were washed and air-dried in a well-ventilated room at Pavilion IV, Institute of Biology, University of the Philippines (Diliman, Quezon City, Philippines). After drying, the leaf samples were homogenized using a blender.

### Concentration and solvent fractionation

Homogenized leaf samples were soaked in ethanol for 48 h. The soluble ethanolic extract was filtered and the filtrate was concentrated *in vacuo* at 40°C using a rotary evaporator (Heidolph, Schwabach, Germany) yielding a concentrated ethanolic crude extract (ECE). ECE was assayed for cytotoxicity by using MTT assay [28] against HCT116 cells. The concentrated ECE was diluted in approximately 200 mL ethanol and was partitioned exhaustively with hexane and ethyl acetate successively. Both hexane soluble extract and ethyl acetate soluble extract were concentrated *in vacuo* at 40°C separately which yielded concentrated hexane extract (HE) and concentrated ethyl acetate extract (EAE). Finally, the two concentrated crude fractions were tested for their cytotoxicity by the MTT assay.

### Determination of band profiles by thin layer chromatography (TLC)

A small amount of the EAE was spotted 5 cm above the bottom edge of the 5 × 7.5 cm TLC plate using a very thin glass tube. The extract was air-dried on the TLC plate which was then placed in eluent in the developing chamber so the bottom of the plate is in the liquid. Several eluents with increasing/decreasing concentration ratio (ranging from 1:9 to 9:1) of chloroform to methanol were prepared to determine the best solvent system that separates components of the mixture in the extract; the 9:1 chloroform-methanol was used as solvent system in column chromatography.

### Gravity column chromatography

EAE was fractionated using gravity column chromatography. The glass column was packed with Silica gel Merck® grade 9385, pore size 60 Å, 230–400 mesh. A 9:1 chloroform-methanol was used as an eluting solvent in the first batch of fractionation to produce column fractions (CF). Increasing gradient concentrations of hexane and ethyl acetate were used in the next batch to produce sub-fractions (SF). An appropriate amount of extract was applied to the top of the column to prevent overloading: 1.0 g of extract to 1 kg of silica gel was packed in the glass column. The eluates (20 mL) were collected in test tubes and separation was monitored by TLC. Those eluates with same TLC profiles were pooled together, concentrated in a rotary evaporator and labeled as chromatographic fractions (CF). TLC profiles of the eluates were visualized by using UV light (100 nm – 315 nm) to detect the presence of aromatic compounds and 10% vanillin-sulfuric acid as spray reagent to detect groups of unsaturated compounds on the TLC plate. Pooled fractions were then subjected to MTT assay. Fractions that showed the highest cytotoxic activity were further fractionated in gravity column chromatography (2 cm in diameter and 37 cm long) through gradient elution. An active isolate was finally purified by high performance liquid chromatography (HPLC) using Silica–C18 as packing material and methanol–water as the mobile phase and was submitted for mass spectrometry to determine the molecular weight and nuclear magnetic resonance (NMR) analysis to determine the possible structure of the compound.

### Cell viability assays

To determine the cytotoxic effects of the crude extract and isolated compound, cellular growth of both untreated and treated cells was monitored using the MTT (4,5-dimethylthiazol-2-yl)-2,5-diphenyltetrazolium bromide) assay [28]. A master dilution plate (MDP) was prepared before subjecting the cells to MTT assay. Each MDP contained *A. loheri* extracts/isolate in varying concentrations ranging from 125–1000 μg × mL^-1^ for crude extracts and 7.8-62.5 μg × mL^-1^ for pure isolates. Cells were harvested and seeded in a sterile, flat-bottom 96-well microtiter plates at a density of 3.5 × 10^5^ mL^-1^, then were incubated for 24 h at 37°C in a humidified incubator with 5% CO_2_. After 24 h incubation, 10 μL from MDP preparation were added to the cells in each well to make final concentrations of 50, 25, 12.5 and 6.25 μg × mL^-1^ for crude extracts and 7.8, 15.6, 31.25, 62.5 μg × mL^-1^ for the pure isolate. The cells were then incubated for 72 h. After incubation, the media was discarded, and 20 mL of MTT were added to each well. The MTT solution was made by dissolving 5 mg MTT (Sigma-Aldrich) per mL phosphate buffer solution. The cells were again incubated for 4 h before adding 150 mL of DMSO (Sigma, USA) to dissolve the formazan crystals. At least two independent tests with three replicates each were performed in this experiment. The absorbance of the formazan crystals was then taken with dual wave-measurement at 570 nm and 620 nm using a Bio-Rad microplate reader (Munich, Germany).

Furthermore, a colorimetric XTT-based assay was applied according to the manufacturer’s protocol. Two independent tests were conducted. Cells grown in cell culture flasks were harvested. A 100 μL aliquot of 2.0 × 10^5^cells/mL were seeded into each well of a 96-well microtiter cell culture plate, and were immediately treated with various concentrations (0.001 to 30 μM) of Maldi 531.2[M + H]^+^, the isolate, with six replicates per dose level. Two sets of control groups were employed: one control group was suspended only in nutrient media while another was suspended in nutrient media containing 0.02% dimethyl sulfoxide (DMSO), the solvent to dissolve Maldi 531.2[M + H]^+^. After incubating for 72 h at 37°C and 5% CO_2_, 50 μL of a mixture of XTT labeling reagent and electron-coupling reagent in sterile phosphate buffered saline (PBS) were added and incubated at 37°C for 4 h. The absorbance of the water-soluble formazan formed was measured at 490 nm and 655 nm using a Tecan microplate reader (Sunrise-Basic Tecan, Crailsheim, Germany) and is directly proportional to the number of living cells in the culture. The viability of the treated cells was compared to the viability of the DMSO-treated cells. Percent growth inhibition (*I%*) was calculated using the formula: *I% = 100 [1-(A*_*t*_*/A*_*c*_*)* where *A*_*t*_ is the absorbance of the Maldi 531.2[M + H]^+^ -treated cells and *A*_*c*_ is the absorbance of the DMSO-treated cells. The IC_50_ concentration was estimated by logarithmic regression [29] where IC_50_ is the inhibitory concentration of Maldi 531.2[M + H]^+^ required to inhibit growth of 50% of the incubated cell population.

### Mitochondrial membrane potential (ΔΨm) analysis

JC-1 is a membrane permeable dye that selectively accumulates in the mitochondria, where it reversibly changes color as membrane potential (ΔΨm) increases over values of about 80–100 mV. JC-1 is widely used for quantifying mitochondrial membrane potential by measuring the fluorescence intensities and is therefore an indicator of initiation of cell apoptosis [30]. To assess the effect of Maldi 531.2[M + H]^+^ on ΔΨm, 10^6^ cells were incubated for 24 and 48 h (37°C, 5% CO_2_) with three doses (0.05, 0.5 and 1.0 μM). Two aliquots of cells were treated with the control drugs: 10 μg/mL doxorubicin and 0.05 μM CCCP (Sigma-Aldrich) and incubated at 37°C and 5% CO_2_. An incubation time of 10 min was sufficient, because of the rapid stimulation of membrane polarization by CCCP. An incubation of more than 10 min with CCCP greatly reduces the fluorescence intensity in the FL2 channel (564–606 nm) converting a red fluorescence signal to a green signal. Afterwards, the cells were stained with JC-1 for 30 min at 37°C and 5% CO_2_. The membrane sensitive JC-1 dye was prepared according to the manufacturer’s recommended concentration of 2.5 μg/million cells/mL from a stock solution of 1 mg/mL prior to use (eBioscience, Germany). The dye was pre-warmed in a 37°C water bath prior to addition to treated and untreated cells. Stained cells were then analyzed by flow cytometry (FACS®Calibur (Becton Dickinson) and CellQuest Pro (Becton Dickinson) analysis software according to the settings suggested in the protocol (AccuriCytometers, St. Ives Cambs, UK).

### Annexin V/FITC binding assay

Annexin V/FITC staining was performed to evaluate apoptosis induced by Maldi 531.2[M + H]^+^. Annexins are calcium-dependent, phospholipid-binding proteins that specifically bind to phosphatidylserine (PS) in the plasma membrane [31]. Under normal physiological conditions, PS is predominantly located in the cytosolic layer of the plasma membrane. Upon initiation of apoptosis, PS is translocated from the cytosolic layer of the plasma membrane to the extracellular layer for recognition and uptake by phagocytes, where it can be detected by fluorescently-labeled Annexin V (eBioScience). Cells were exposed to the active isolate (0.05, 0.5, 1.0 μM) and incubated for 24 h at 37°C and 5% CO_2_. For positive controls, cells were treated with 10 μg/mL doxorubicin (Sigma-Aldrich) and 10 μM camptothecin separately and were also incubated for 24 h at 37°C and 5% CO_2_. The annexin-V assay was performed according to the protocol provided in the annexin V-FITC detection kit (eBioScience). Briefly, 5.0 × 10^5^ treated cells were re-suspended in 50 μL of 1× annexin-staining buffer containing 10 mM HEPES, 140 mM NaCl, and 2.5 mM CaCl_2_ (pH 7.4) and incubated in the dark with 2.5 μL of annexin V conjugate for 15 min at 25°C. After incubation, the final volume of each sample was adjusted to 500 μL followed by the addition of 1.25 μL of 1 mg × mL^-1^ propidium iodide (PI). Apoptotic cells were analyzed using a FACS®Calibur (Becton Dickinson, Heidelberg, Germany) and CellQuest Pro (Becton Dickinson) analysis software. Excitation and emission settings were 488 nm, 515–545 nm (FL1 channel) for annexin V-FITC and 564–606 nm (FL2 channel) for PI.

### Statistical analysis

Data were expressed as mean ± SEM of two independent tests. JC-1 and annexin V-FITC fluorescence intensity data were subjected to one-way analysis of variance followed by Tukey’s multiple comparison test. A probability of less than or equal to 0.05 was considered statistically significant to reject the null hypothesis.

## Results

### Bioactivity-guided isolation of an active fraction from *A. loheri*

Approximately 15.23 g (1.96%) of ethyl acetate crude extract were obtained from 777 g of dried ground leaf samples of *A. loheri* after solvent partitioning. Cytotoxicity of the extracts was tested towards HCT116 by the MTT assay. The ethyl acetate fraction was cytotoxic exhibiting an IC_50_ concentration of 5.47 μg/mL. Ethyl acetate extract was subjected to gravity column chromatography utilizing silica gel-60 as stationary phase packed in 3.5 cm diameter, 37 cm long glass column, and 9:1 chloroform-methanol as mobile phase. This chromatographic separation yielded 10 column fractions, where CF1 revealed the highest cytotoxicity (IC_50_ of 3.82 ± 0.02 μg/mL). For comparison, doxorubicin was used as control drug and revealed an IC_50_ of 3.74 ± 0.14 (Table 1). Further gradient fractionation of CF1 yielded 11 sub-fractions (SF) whose IC_50_ values are shown in Table 2. SF9 was the most cytotoxic one. Therefore, this fraction was subjected to HPLC to further purify the active compound. Four distinct peaks were generated. The eluates collected from peaks 1, 2 and 3 were colorless while peak 4 was greenish in color. Cell viability test results after cytotoxicity assay are shown in Table 3.

**Table 1:** **IC**_**50 **_**values of gravity column chromatographic fractions of *Aglaia loheri *** **extracts run in silica gel-60 and 9:1 chloroform: methanol tested for toxicity against HCT 116**

**Column fraction (CF)**	**IC**_**50 **_**(μg/mL)**
**1**	**3.82 ± 0.02**
2	3.84 ± 0.07
3	3.88 ± 0.10
4	3.86 ± 0.12
5	3.98 ± 0.08
6	3.84 ± 0.09
7	5.07 ± 0.16
8	26.60 ± 2.60
9	13.50 ± 0.54
10	NLI
Doxorubicin	3.74 ± 0.14

NLI: No Logarithmic Interpolation indicating absence of toxicity.

Bold Texts: Most cytotoxic fraction of *Aglaia loheri* extracts.

**Table 2:** **Gravity column chromatographic sub-fractions (SF) of *A. loheri *** **CF1 run in silica gel-60 and various gradients of increasing amount of ethyl acetate**

	**Gradient**	
**Sub-fractions (SF)**	**(Percent ratio of eluent: hexane to ethyl acetate)**	**IC50**
		**(μg/mL)**
1	85:15	NLI
2	83:17	42.06 ± 0.67
3	81:19	35.17 ± 4.63
4	78:22	31.61 ± 6.52
5	75:25	40.55 ± 7.24
6	71:29	NLI
7	67:33	NLI
8	63:37	NLI
**9**	**59:41**	**3.96 ± 0.07**
10	55:45	4.98 ± 0.50
11	50:50	11.92 ± 1.29
Doxorubicin		4.31 ± 0.06

NLI: No Logarithmic Interpolation indicating absence of toxicity.

Bold Texts: Most cytotoxic sub-fraction of CF1 (Table 1).

**Table 3:** **IC**_**50 **_**values of eluates collected from individual HPLC peaks**

**Peaks**	**Amount collected (mg)**	**IC**_**50 **_**(μg/mL)**
1	0.2	5.25 ± 0.54
2	0.3	21.35 ± 0.67
**3**	**2.0**	**4.52 ± 0.71**
4	0.4	5.57 ± 0.36
Doxorubicin		4.10 ± 0.28

Bold Texts: Most cytotoxic HPLC fraction from SF9 (Table 2).

### Chemical characterization of the bioactive *A. loheri* fraction

**Figure 1:**
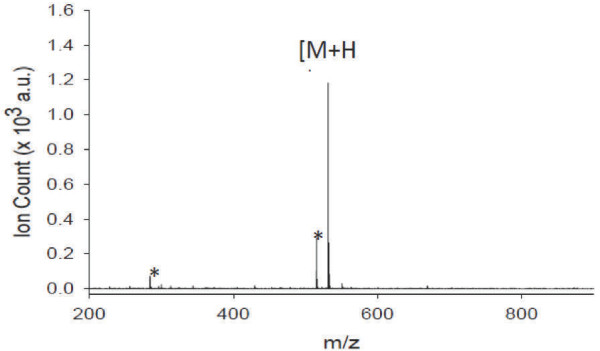
**MALDI MS spectrum of an isolate from *Aglaia loheri.*** [M + H]^+^ = molecular ion peak, m/z 531.2; * denotes background peaks from MALDI matrix.

Bioactivity-guided fractionation of a crude extract of *A. loheri* leaves led to the isolation of a bioactive component (peak 3), an amorphous white powder of approximately 2.0 mg (total yield = 1.3%) which exhibited a molecular ion peak at *m/z* 531.2[M + H]^+^ (Figure 1), which was termed Maldi 531.2[M + H]^+^.

The chemical shift values and their assignment to Maldi 531.2[M + H]^+^ was based on its ^1^H NMR spectrum (Table 4). The ^1^H NMR spectrum indicated that Maldi 531.2[M + H]^+^ contains aromatic rings (δ 6.7-7.9 ppm), a phenolic group (δ 4.54 ppm), methoxy groups (δ 3.61-3.66 ppm), and several aliphatic chain structures (δ 1.87-3.61 ppm). As can be deduced from the one-dimensional NMR analysis, the probable structure of Maldi 531.2[M + H]^+^ may be an aromatic (phenolic) ester.

**Table 4:** ^**1**^**H NMR spectral data of Maldi 531.2[M + H]**^**+**^

**PPM**	**Multiplicity**	***J coupling***	**TYPE**
1.87	m		NHCO phenyl
2.70	s		-OCO-alkyl or phenyl
3.19	m		N-propyl CH2OH
3.42	m		Methyl CH3OH
3.61	m		CH2-OH
3.62	s	-	-OCH3
3.66	s	-	-OCH3
3.72	m	-	-OCO-CH3
3.82	d	6.72	-OCO- phenyl or methyl
3.97	dd	7.92; 6.24	Isopropyl CH-OH
4.23	d	14.22	-OCO-phenyl or n-propyl
4.58	bs	-	Phenolic –OH?
5.49			-OCO-phenyl
6.17	d	1.92	Aromatic H
6.28	d	1.80	Aromatic H
6.61	dd	8.24; 3.00	Aromatic H
6.89	d	6.90	Aromatic H
6.99	m		Aromatic H
7.03	m		Aromatic H
7.11	dd	8.94; 3.00	Aromatic H
7.90	s	-	Aromatic H

s: singlet; bs: broad singlet; d: doublet; dd: doublet of doublet; m: multiplet.

### Cytotoxicity of Maldi 531.2[M + H]^+^ towards sensitive and multidrug-resistant tumor cells

**Figure 2:**
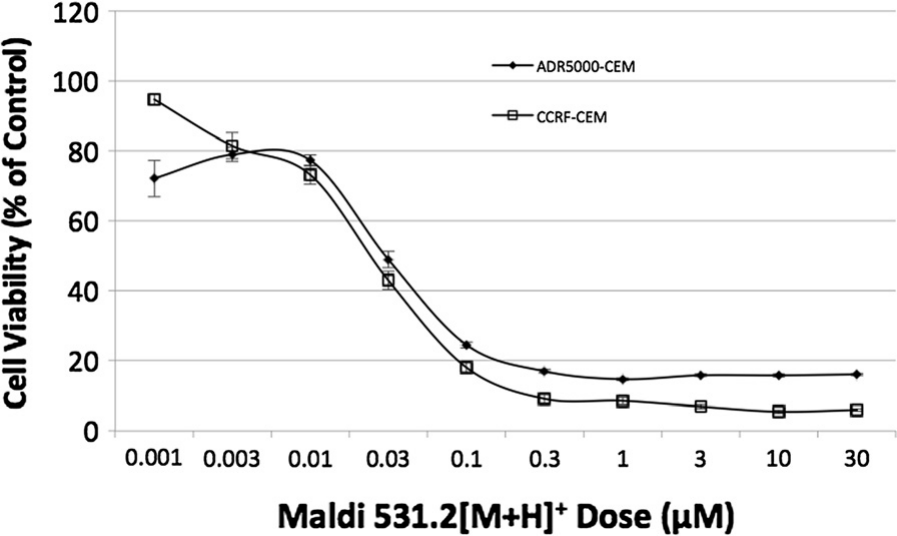
**The effect of Maldi 531.2[M + H]**^**+ **^**on CCRF-CEM and CEM/ADR5000 cells determined by XTT assay after incubating the cells with the isolate for 72 hr. in a humidified environment.** Values are means ± SEM of six replicates each of two independent experiments.

Figure 2 shows the effects of Maldi 531.2[M + H]^+^ on the viability of CCRF-CEM cells and their multidrug-resistant sub-line, CEM/ADR5000. The active principle reduced mean cell viability of the two human leukemic cell types in a dose-dependent manner. A significant decrease in cell viability was observed at 0.03 μM and this further decreased with increasing concentration. Few cells survived at a dose range of 0.3 to 30 μM. The IC_50_ of Maldi 531.2[M + H]^+^ on CCRF-CEM cells was 0.02 μM and on CEM/ADR5000 cells 0.03 μM, indicating that CEM/ADR5000 cells were only 1.5-fold cross-resistant to Maldi 531.2[M + H]^+^. For comparison, CEM/ADR5000 cells were more than 1000-fold resistant towards doxorubicin compared to parental CCRF-CEM cells [32]. Maldi 531.2[M + H] + was also tested against HCT 116 cells yielding a mean IC_50_ of 3.9 μg/mL (data not shown). However, an MDR suspension cell line and its corresponding sensitive line, were used for the later experiment due to the ease of maintaining and handling suspension cell line over that of adherent cell line which need constant trypsinization. Although it is important that the cytotoxic agent be determined to be specific only to cancer cells, it is an established fact that many of the presently used cancer chemotherapeutic agents are not specific against tumor/ malignant cells only. They also affect fast dividing normal cells such as those in the blood, in the digestive tract and other organs leading to a number of side effects like vomiting, hair loss, and the like. Hence, a number of studies that seek to mitigate these effects on normal cells such as on drug delivery specifically targeting cancer cells are being actively explored. We also suggest this as an offshoot of this study as part of the recommendation or future directions or follow up studies.

**Figure 3:**
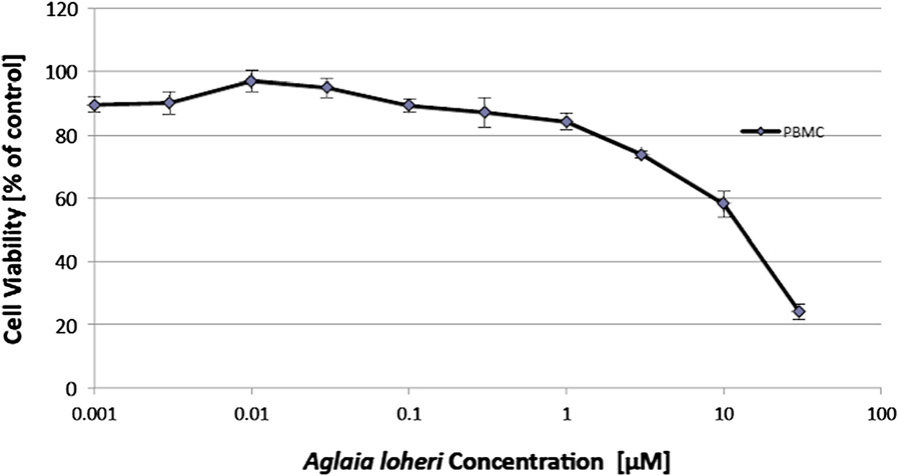
**Growth inhibition of Maldi 531.2[M + H]**^**+ **^**toward peripheral blood mononuclear cells (PBMC) activated with 10 μg/mL PHA.** Viability of PBMC was determined by XTT assay after incubating the cells with the isolate for 72 hr. in a humidified environment (37°C and 5% CO_2_). Values are means ± SEM of six replicates each of two independent experiments.

In connection with this, a cell viability test was performed separately using PHA-activated peripheral blood mononuclear cells (PBMC). After exposing the cells with various concentrations of Maldi 531.2[M + H]^+^, it was found to be cytotoxic (IC_50_: 50.86 *μ*M) as seen in Figure 3. However, despite its cytotoxic effects on PBMC, the level of toxicity is much less than its effects toward CCRF-CEM and ADR5000/CEM cells.

### Quantitation of intrinsic mitochondrial membrane potential by flow cytometry

**Figure 4:**
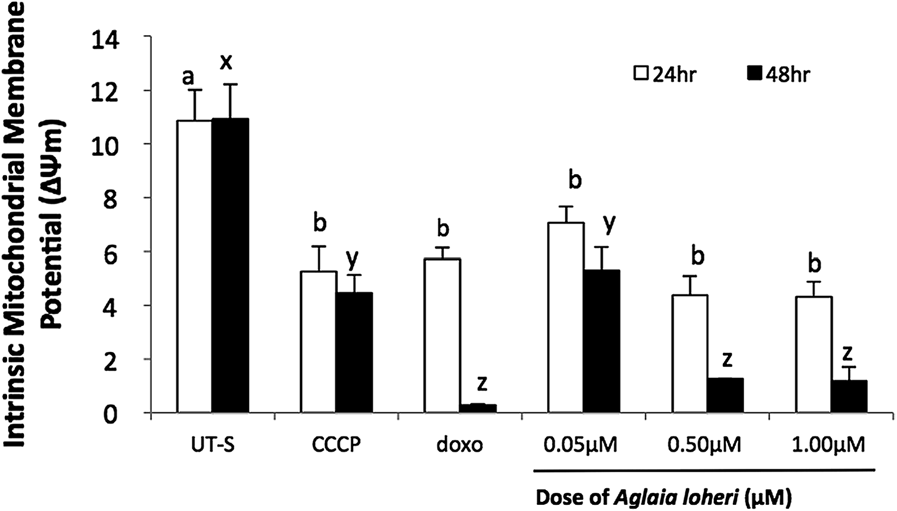
**Determination of mitochondrial membrane potential (ΔΨm) in multidrug-resistant CEM/ADR5000 cells after 24 and 48 h exposure to Maldi 531.2[M + H]**^**+**^**.** Data are means ± SEM of the three replicates of two independent experiments; P < 0.05. Untreated cells served as negative control and 10 μg/mL doxorubicin (doxo) and 0.05 μM carbonyl cyanide 3-chlorophenyl hydrazone (CCCP)-treated cells as positive controls. Significant differences between are indicated by letters. After 24 hr treatment, mitochondrial membrane potential (ΔΨm) of untreated cells (UT-S), indicated by subset “a” is significantly different from the CCCP and Maldi 531.2[M + H] + treated cells as indicated by subset “b”. Values for CCCP and Maldi 531.2[M + H] + treated cells don’t show significant differences from each other as belong to the same subset “b”. Mitochondrial membrane potential (ΔΨm) of untreated cells (UT-S), indicated by subset “x” is significantly different from the CCCP and Maldi 531.2[M + H] + treated cells as indicated by subset “y” and “z” for 48 hrs. Values of same subset at non-significant from each other.

Figure 4 shows the mitochondrial membrane potential of CEM/ADR5000 cells treated for 24 and 48 h with various doses of Maldi 531.2[M + H]^+^. A significant ΔΨm reduction was observed in cells treated with the active principle in a dose-dependent manner 24 h after treatment compared with untreated cells. A further significant reduction of ΔΨm with Maldi 531.2[M + H]^+^-treated cells was observed after 48 h. However, reduction in Maldi 531.2[M + H]^+^-treated cells was not significantly different from the positive controls doxorubicin and CCCP after 24 h, but significantly decreased in those cells treated with 0.5 and 1.0 μM Maldi 531.2[M + H]^+^ including those of doxorubicin-treated cells after incubation for 48 h.

### Annexin-FITC binding assay

**Figure 5:**
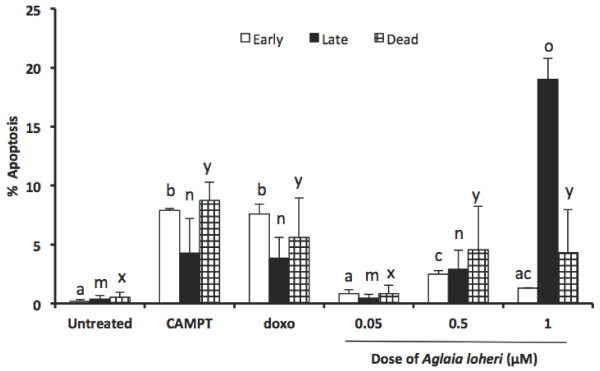
**Measurement of apoptosis induction by Maldi 531.2[M + H]**^**+**^**in CEM/ADR5000 determined by incubation with annexin V.** Data are means ± SEM of three replicates of two independent experiments with reference to their controls: Untreated cells served as negative control; 10 μM camptothecin (CAMPT) and 10 μg/mL doxorubicin (doxo) - treated cells as positive control. Significant differences in apoptotic activity between treatments are indicated by various groups of subsets: Early - “abc”; late apoptosis - “mno” and dead cells – “xy”. Cells treated with 0.05 μM Maldi 531.2[M + H]^+^ did not show significant apoptotic activity compared to negative control cells in all stages but significantly different from cells treated with the positive controls and those cells treated with 0.5 and 1.0 μM Maldi 531.2[M + H]^+^ (P < 0.05). However, early apoptosis in cells treated with 0.05 μM Maldi 531.2[M + H] + is not significantly different from early apoptosis in cells treated with 1.0 μM but significantly different from 0.5 μM Maldi 531.2[M + H] + treated cells. Bars of same subsets are not significantly different from each other.

Apoptosis was induced by Maldi 531.2[M + H]^+^ in a dose-dependent manner (Figure 5). Compared with untreated cells, early apoptosis did not significantly differ in all treatments, but significantly increased in late apoptosis in a dose-dependent manner. Compared with camptothecin and doxorubicin-treated cells Maldi 531.2[M + H]^+^ reduced the occurrence of early apoptotic, but not late apoptotic and dead cells over time.

## Discussion

Plant extracts usually contain many secondary metabolites with varying bioactivities [33]. In the present investigation, we report the isolation of a cytotoxic component of *A. loheri*, termed Maldi 531.2[M + H]^+^. The cytotoxic properties of genus *Aglaia* was previously recognized by several groups [34]. Therefore, chemical studies of the constituents of *Aglaia* focused primarily on its toxic components resulting in the structure elucidation of numerous phytochemical compounds [34]. To date, *Aglaia* still represents a promising and rich source for novel compounds. The isolation and purification of Maldi 531.2[M + H]^+^ from *A. loheri* proved to be difficult, because the isolated active component, was only present in very low amounts. Despite this limitation, we were able to investigate the bioactivity of Maldi 531.2[M + H]^+^ towards cancer cells. Based on mass spectrometry analyses, this compound does not reveal any similarity with other compounds isolated from other *Aglaia* species [19,20]. As shown in Tables 5 and 6, the molecular ion peaks of other *Aglaia* compounds are distinct from that of our *A. loheri* isolate with *m/z* 531.2[M + H]^+^, indicating that this represents a still unknown compound at least for the genus *Aglaia*.

**Table 5:** **List of unknown compounds previously isolated from *Aglaia edulis *** **tested and found active against several human cancer cell lines (Kim et al., 2006)**[19]

**Compound number**	**m/z**	**Elemental formula**	**Assigned name of the compound**
1	^	^	Aglaroxin A
2	584.1875*	C_31_H_31_NO_9_Na*	Aglaroxin A 1-O-Acetate
3	614.1999	C_32_H_33_NO_10_Na	3’-methoxyaglaroxin A-1-O-Acetate
4	693.2421	C_37_H_38_N_2_O_10_Na	19,20-dehydroedulisone A
5	*	*	Edulirin A
6	**	**	Edulirin A-10-O-Acetate
7	665.2469	C_36_H_38_H_2_O_9_Na	9,20-dehydroedulirin A
8	667.2608	C_36_H_40_N_2_O_9_Na	Isoedulirin A
9	***	***	Isoedulirin B
10	316.1225	C_17_H_20_N_2_O_2_S	Aglamide A
11	335.1079	C_17_H_20_N_2_O_3_SNa	Aglamide B
12	323.1732	C_18_H_24_N_2_O_2_Na	Aglamide C
13	254.1151	C_14_H_17_NO_2_Na	Aglamide D

^ - known compound; * - similar to compound 2; ** - almost identical to compound 5; *** - similar to compound 8.

**Table 6:** **List of compounds previously isolated from *Aglaia foveolata *** **tested and found active against cancer (Salim et al., 2007)**[20]

**Compound number**	**m/z**	**Elemental formula**	**Assigned name of the compound**
**1**	^	^	Silvestrol
**2**	675.2682*	C_38_H_40_N_2_O_8_Na*	^^
**3**	*	*	^^
**4**	*	*	^^
**5**	673.2528	C_38_H_38_N_2_O_8_Na	^^
**6**	661.2529	C_37_H_38_N_2_O_8_Na	^^
**7**	^	^	Pyramidatine (bisamide)
**8**	559.3606	C_13_H_52_O_7_Na	^^

^ - known compound; ^^ - no assigned compound name; * - similar compounds.

The isolate could possibly be related to bisamides and flavaglines isolated by Kim et al. [19] and Salim et al. [20] based on their cytotoxic and anticancer activities. Cytotoxic activities were also observed in other isolated compounds from *A. tenuicaulis* and *A. forbesii* such as amide esters [14,17], which also demonstrated bioactivity, *e.g.* anti-inflammatory and antimicrobial [35], antiviral [17], and anticancer activities [36].

Phenolic esters inhibit carcinogenesis and reduce progression of cancer. Understanding the molecular basis of their anticancer properties based on structure and activity is important for the rational design of novel chemotherapeutic agent [37]. Maldi 531.2[M + H]^+^ is a phenolic derivative. Structure, lipophilicity and antioxidant properties of the compounds are major factors affecting the cytotoxicity of the compound due to the presence of a number of hydroxyl (−OH) ring components and by the length of the alkyl esters within the structure [37]. Lipophilicity goes with the number of alkyl esters, *i.e.* the longer the alkyl esters in the molecule, the greater its anticancer effect [37]. For example, propyl esters are more effective against tumor cells than methyl esters. In addition, –OH groups are responsible for the antioxidant property in phenolic compounds and their methylation diminishes their cytotoxicity [38]. However, lipophilicity and antitumor activity were difficult to correlate, because other structural characteristics may also account for the cytotoxicity of Maldi 531.2[M + H]^+^. This includes the position of the phenolic substituents such as aromatic rings, carbons, carboxyl group (C = O), and the alkyl chains.

Apoptosis is a fundamental, energy-dependent biological mechanism [31,39], which can be triggered by a diverse array of stimuli, including phytochemicals [40,41]. The reduction of ΔΨm by Maldi 531.2[M + H]^+^ in the present study strongly indicates initiation of apoptotic events and inhibition of cell proliferation by dissipation of the mitochondrial membrane potential. Mitochondria are dynamic organelles, as they constantly produce ATP [42]. A decrease of the mitochondrial membrane potential results in loss of mitochondrial function as it is an important component of the electrochemical gradient that is generated across the inner mitochondrial membrane during electron transport and oxidative phosphorylation [43]. Thus, ΔΨm is used as an indicator of cell integrity [23], which is critical for normal function of mammalian cells, maintaining the function of mitochondrial proteins in the production of ATP and reactive oxygen species (ROS). Alterations of the mitochondrial membrane potential are indicative of changes in mitochondrial metabolic activity (Acton 2004) leading to cell cycle arrest and apoptosis [44].

Two biochemical assays were used to establish apoptosis: annexin V and JC-1 mitochondrial membrane potential assay. The results of these two assays are sufficient to establish the occurrence of apoptosis. JC-1 assay evaluates extent of apoptosis through an end point which is the loss of the mitochondrial membrane potential reflected by the entry and aggregation of the dye inside the mitochondrial matrix. This event presupposes that cytochrome C has been released, an event associated with the loss of the mitochondrial membrane potential. These two events however may not always go together since it has been shown that loss of mitochondrial membrane potential is not necessarily required for cytochrome c release [45].

In the case of the cytotoxicity effect of Maldi 531.2[M + H] + as shown in cell viability tests and annexin V-FITC binding assay, the difference in IC_50_ values with XTT at 0.02-0.03 uM and the non-significant difference of cytotoxic effect at 0.05 uM with annexin V FITC binding assay for apoptosis, relative to untreated cells, is the time of observation. The cytotoxicity assay required 3 days (72 hours) of incubation with varied concentrations of the drug/ natural product to determine the growth inhibitory concentration which is the IC_50_ where 50% of the population of cells have died already at a range of concentration of 0.02 uM-0.03 uM for CCRF-CEM and CEM/ADR5000 respectively. In the case of apoptosis, observation time was only 24 hours after treatment purposely to check only the ability of Maldi 531.2[M + H]^+^ to “initiate” apoptosis. The optimum number of hours for Maldi 531.2[M + H] + must not exceed 24 hours. If cells were incubated longer than 24 hours with Maldi 531.2[M + H]^+^, i.e. after 32 or 48 hours, all cells were dead which may significantly affect flow cytometric reading for apoptosis. Annexin V-FITC binding assay was used to detect PS exposure on the membranes of apoptotic cells with high affinity. Annexin V binding assay detects apoptotic cells significantly even earlier than detections based on alteration in gross DNA structures [31].

Although huge efforts have been undertaken to identify P-glycoprotein inhibitors to overcome multidrug resistance [46,47], clinical trials failed as yet [47,48]. Another concept is to use non-cross-resistant cytotoxic compounds to kill multidrug-resistant tumors. Remarkably, CEM/ADR5000 cells did not reveal considerable cross-resistance to Maldi 531.2[M + H]^+^ (1.5-fold), although CEM/ADR5000 cells were highly resistant to anthracyclines, *Vinca* alkaloids, taxanes and other established drugs as previously reported [32]. This is a remarkable result, since it implies that Maldi 531.2[M + H]^+^ might be suitable to kill refractory tumors, which are resistant to standard chemotherapy.

## Conclusion

In conclusion, Maldi 531.2[M + H]^+^ showed considerable activity against sensitive and multidrug-resistant tumor cells by dissipation of the mitochondrial membrane potential and induction of apoptosis. *A. loheri* and Maldi 531.2[M + H]^+^ may be promising for further drug development. Further studies are required to unravel the full potential of Maldi 531.2[M + H]^+^ as novel drug candidate for cancer therapy.

## Abbreviations

ALL: Acute lymphoblastic leukemia; CCCP: Carbonyl cyanide 3-chlorophenylhydrazone; CCRF-CEM: Sensitive human leukemia cells; CEM/ADR5000: Multidrug resistant leukemia cells; CF: Column fraction(s); EAE: Ethyl acetate extract; ECE: Ethanolic concentrated extract; HCT116: Human Colon cancer cell line; HE: Hexane extract; HPLC: High liquid performace chromatography; MDP: Master dilution plate; MTT: 3-(4,5-dimethylthiazol-2-yl)2,5-diphenyltetrazolium bromide); NMR: Nuclear magnetic resonance; PBMC: Peripheral blood mononuclear cells; PHA: Phytohemagglutinin; PI: Propidium ioodide; PS: Phosphatidylserine; SF: Sub-fractions of CF; TLC: Thin layer chromatography; XTT: 2,3-bis(2-methoxy-4-nitro-5-sulfophenyl)-2H-tetrazolium-5-carboxalnilide).

## Competing interests

The authors declare that there are no competing interests involved in this research study.

## Authors’ contributions

SJ and TE planned and supervised the research work (SJ in the Philippines; TE in Germany). SJ helped in collection of plant materials, funded (through her research grant) the isolation of Maldi 531.2[M + H)^+^; TE funded expenses for all assays in the laboratory (Germany); ED performed the isolation of Maldi 531.2[M + H]^+^ (in the Philippines) and all assays in Germany. All authors read and approved the final manuscript.

## Pre-publication history

The pre-publication history for this paper can be accessed here:

http://www.biomedcentral.com/1472-6882/13/286/prepub
